# Digital teaching and assessment of psychomotor skills of the clinical head and neck examination during COVID-19 pandemic

**DOI:** 10.1007/s00405-023-07998-8

**Published:** 2023-05-12

**Authors:** Theresa Lüdke, Marie-Luise Polk, Susanne Günther, Anne Kluge, Thomas Zahnert, Marcus Neudert

**Affiliations:** Department of Otorhinolaryngology Head and Neck Surgery, TU Dresden Faculty of Medicine and University Hospital Carl Gustav Carus, Fetscherstrasse 74, 01307 Dresden, Germany

**Keywords:** Digital teaching, Psychomotor skills, Digital assessment, COVID-19

## Abstract

**Purpose:**

During COVID-19, a fully digital course was established for teaching and assessing the psychomotor skills of clinical head and neck examination. Influence of different digital teaching formats was investigated.

**Methods:**

The students (*n* = 286) received disposable instruments, a manual, and instructional videos for the examination. 221 students additionally received 45 min of interactive teleteaching. After 5 days of practice, all students were required to submit a video of their examination and report their spent practice time. The assessment was carried out using a checklist which was already established in presence teaching.

**Results:**

The average score achieved by digital teaching was 86%. Previously published data show that presence teaching achieved 94%. With a teleteaching unit the total score was significantly better than without (87% vs 83%)*.* Teleteaching leads to a significant positive correlation between practice time and total score. Without teleteaching there is a negative correlation. After the same practice time, presence teaching leads to better total scores than digital teaching.

**Conclusion:**

Digital teaching and assessing of a complex psychomotor skill is possible. Interactive teaching methods increase learning success. Nevertheless, presence teaching seems to be better at teaching these skills. The results can provide a basis for developing hybrid teaching models.

## Introduction

Curricular teaching has been facing new directions since 2020 due to the COVID-19 pandemic. The rapid conversion of previously established presence teaching to digital teaching formats poses several challenges for teachers as well as learners. On the one hand, the spatial separation leads to the loss of direct physical contact and thus often to a greater hurdle for direct questions and comments from the learner. Likewise, the instructor is unable to evaluate the learner’s reactions and emotions and to survey a broad crowd as in a seminar or lecture hall. Another major challenge is the examination format, for which there were no legal requirements in the digital domain at the beginning of the COVID-19 pandemic. For example, there was a lack of good and legal programs to verify examinees’ personal data. In recent months, however, there has been tremendous progress. In the meantime, there are numerous digital exam formats that are legally available.

These challenges are multiplied in the case of teaching psychomotor skills, which play a crucial role in medical studies. In addition to the definition of pure learning objectives, a paradigm shift has also occurred as a result of competency-based learning, as now the focus is not only on what is taught, but on the outcome—the competency to be mastered at the end of the learning process. For this purpose, the National Competence-Oriented Catalog of Learning Objectives in Medicine (NKLM) [1] is considered the orientation recommendation for medical faculties in Germany. Outside of pandemic situations, the training program for students includes bedside teaching and skills lab units in high numbers of hours. Due to the COVID-19 pandemic, direct patient contact was not possible in 2020 and 2021. Contact with other fellow students was restricted by strict regulations at the beginning of the COVID-19 pandemic. Therefore, new alternatives for teaching practical skills had to be created.

In Otorhinolaryngology, Head and Neck Surgery (ORL-HNS), the requirements for teaching and assessing the practical skill of clinical head and neck examination (CHNE) in presence format had been established in previous years. A clear definition of the learning objective was made via the operationalization of the CHNE and the creation of a checklist-based assessment [2, 3]. Using the checklist, the learning curve was measured during the daily training of the CHNE of the students and it was shown that there was an improvement in psychomotor skills with an increase in training sessions [4]. The CHNE can, therefore, be considered a very well characterized psychomotoric skill in medical student education. The learning process and progress are quantified formatively and summatively. It is therefore an excellent tool for drawing comparisons between the new digital teaching format and traditional presence face-to-face teaching.

The present study aims to answer the following questions:

- Can psychomotor learning content be conveyed by means of digital teaching formats and learnt predominantly through self-study?
- What is the impact of interactive teleteaching on the student’s exam success?
- Are the exam results of digital CHNE course comparable to those of presence CHNE course?

## Methods

### Study population

The study was prospectively conducted on all 10th semester (fifth and final year) medical students (*n* = 286) during the summer semester of 2020. Each week, a group of approximately 40 students completed the full digital CHNE course.

### Course of study

**Fig. 1:**
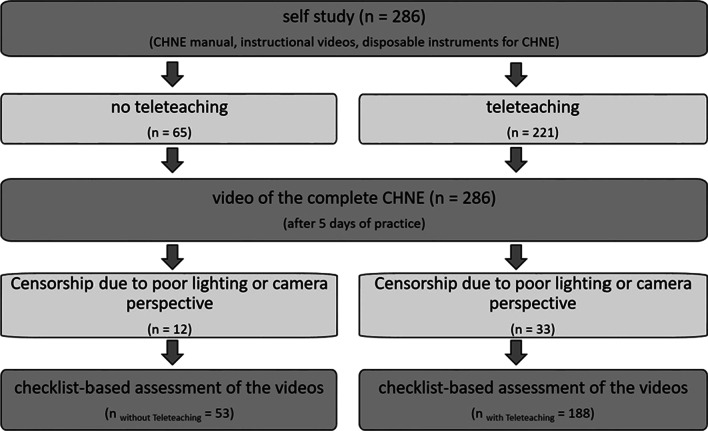
Study design, clinical head and neck examination (CHNE)

At the beginning of the CHNE course, all students (*n* = 286) were given disposable instruments for CHNE (nasal speculum, ear funnel, two wooden mouth spatula, dental mirror). The students had to organise their own headlamp (e.g. bicycle light. Students had to replace the Frenzel goggles and tuning fork with individual items from their own household. Using a paper-based manual as well as an instructional video, the CHNE was presented to the students. The correct performance and sequence of the single examination steps were specifically taught using the operationalised of the CHNE [2]. The students were encouraged to learn the CHNE on their own using the digitally provided teaching materials. For this purpose, they had to find a contact person from their private environment with whom unrestricted contact was possible under pandemic conditions. Students were divided into two groups. One group (*n* = 65) received the previously mentioned teaching materials only (group without teleteaching (GwoTT)). The other group (*n* = 221) received additional livestream teleteaching allowing direct interaction with the tutor (group with teleteaching (GwTT)). An ORL senior physician demonstrated the CHNE via livestream teleteaching (45 min). Questions from the students were also answered during this session and detailed manual instructions were given on student’s demands. After five days of practice, all students were required to send in a video of their complete CHNE. Videos that could not be evaluated due to poor lighting or camera perspective were excluded from the analysis (12 without teleteaching, 33 with teleteaching). In total, 53 videos with teleteaching and 188 videos without teleteaching were evaluated. All students also had to report their practice time (in minutes) for their CHNE when submitting the video. The study design is shown in Fig. 1.

### Measuring instrument

The videos were evaluated by the same ORL physician using the previously described checklist-based assessment [2]. All six ORL subexaminations (otoscopy, anterior rhinoscopy, oral inspection, posterior rhinoscopy, indirect laryngoscopy, general head and neck examination) were analysed. The relative score achieved was given as a percentage in the following (100 = 100%).

### Comparison with the CHNE taught in presence

In 2011, Kemper et al. [2], and in 2020, Polk et al. [4], the presence CHNE course was evaluated using the same standardized checklist as in this study. Kemper et al. [2] published the total scores of CHNE of all students in 2011 during presence teaching. The exam took place after a 5-day presence CHNE-course. In addition to the published [2] total scores of 166 students, we have the data of all students from 2011 (*n* = 261) and the scores of all subexaminations. Using this data record, the digital teaching could be compared with the presence teaching.

In 2020, Polk et al. [4] published the learning curve of CHNE of presence teaching. The presence CHNE course consisted of an initial demonstration by an ORL physician (45 min) followed by 5 days of training (90 min on day 1, 45 min each on days 2–5). The practical examination was performed on day 5 [4]. At the end of each internship day, the learning progress of a total of 48 students was statistically evaluated by a checklist-based assessment. The overall performance [2] as well as the learning curves [4] were used to compare digital teaching and the traditional presence setting.

### Statistical analysis

IBM SPSS Statistics 24 (Fa. SPSS Inc, Chicago/IL, USA) and Microsoft Excel 2010 (Fa. Microsoft Corporation, Redmond/WA, USA) were used to analyse the results. Statistical analysis was performed using the *t* test for independent samples. Pearson correlation was used to calculate the correlation between practice time and total score. A significance level of *p* < 0.05 was defined for all analyses. All values are given as mean value in percentage ± standard deviation (SD).

## Results

### The impact of digital teaching with and without teleteaching

**Fig. 2:**
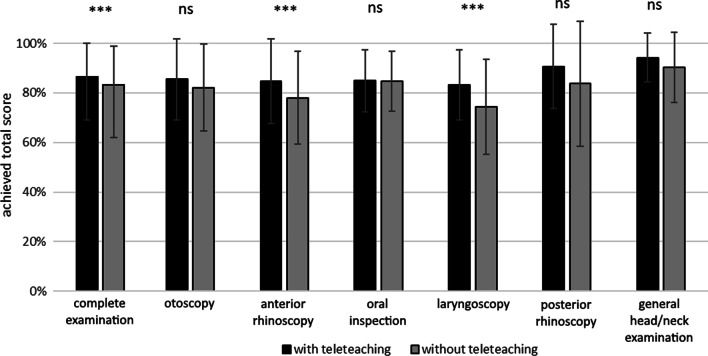
Mean scores and standard deviation of complete clinical head and neck examination and subexamination with and without teleteaching. *ns* not significant, ***p < 0,001

The average of the achieved score of all 241 evaluated videos is 86.1 ± 9.2%. GwoTT achieved 83.0 ± 10.8%, whereas the GwTT was significantly better (87.0 ± 8.5%, *p* = 0.005) (Fig. 2).

The mean values as well as the SD were also calculated for the subexaminations of the CHNE (Fig. 2). The GwTT achieved more points on average in all subexaminations than the GwoTT. However, no significant differences were shown between the GwTT and GwoTT for otoscopy (85.6 ± 16.5 GwTT vs. 82.2 ± 17.6 GwoTT), oral inspection (84.9 ± 12.5 GwTT vs. 84.8 ± 12.1 GwoTT), posterior rhinoscopy (90.6 ± 17.0 GwTT vs. 83.7 ± 25.2 GwoTT), and head and neck examination (94.2 ± 10.0 GwTT vs. 90.2 ± 14.2 GwoTT).

For anterior rhinoscopy and laryngoscopy, the GwTT (84.7 ± 17.1 and 83.3 ± 14.1) reached significantly (*p* < 0.05) higher scores than the GwoTT (78.0 ± 18.7 and 74.4 ± 19.2).

### Correlation of total score and practice time

Seven students did not report their practice time and their results were excluded from this subexamination.

**Fig. 3:**
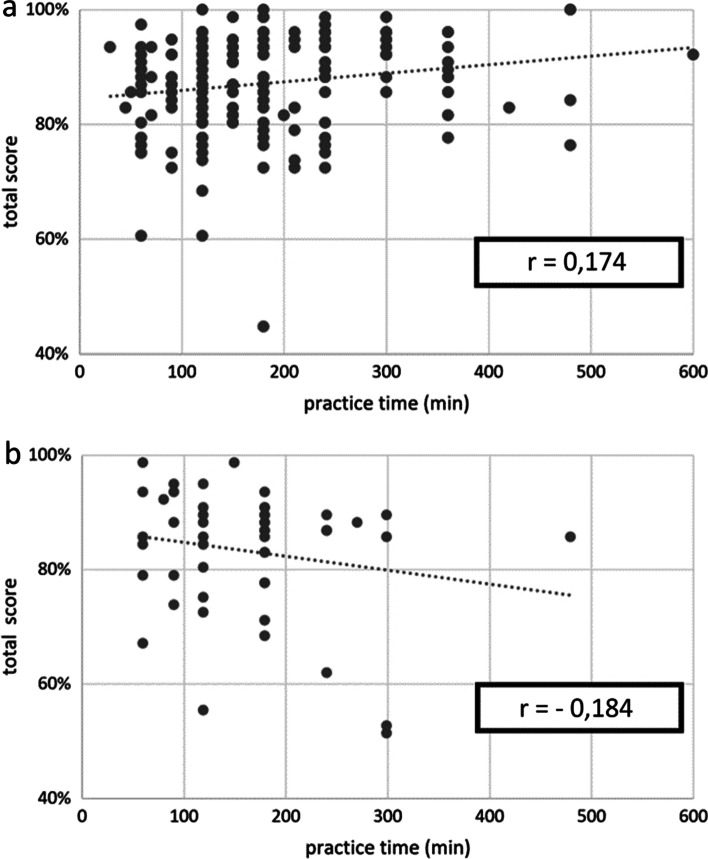
correlation between practice time and total score **a** with teleteaching, **b** without teleteaching

In the GwTT, the evaluation of 181 videos showed a low but significant (*p* = 0.019) positive correlation between practice time and total score (*r* = 0.174), (Fig. 3a). The mean value of practice time is 177 min (minimum: 30 min, maximum: 600 min).

In GwoTT, the 51 cases showed a negative correlation between practice time and total score (*r* = − 0.184). However, this trend was not significant (*p* = 0.197), (Fig. 3b). The mean value of practice time is 165 min (minimum: 60 min, maximum: 480 min).

### Comparison of digital teaching and analog presence teaching

**Fig. 4:**
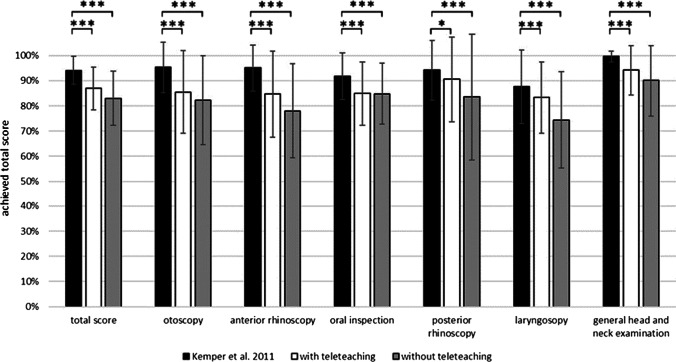
Comparison of presence teaching and digital teaching of the clinical head and neck examination. ***p < 0.001, *p < 0.05

Comparing the data of digital teaching with the data of presence teaching from 2011 (*n* = 261), show that presence teaching is superior to digital teaching in both the overall score and all subexaminations. Presence teaching leads to significantly better results than digital teaching (Fig. 4). These data are not corrected to the practice times.

**Fig. 5:**
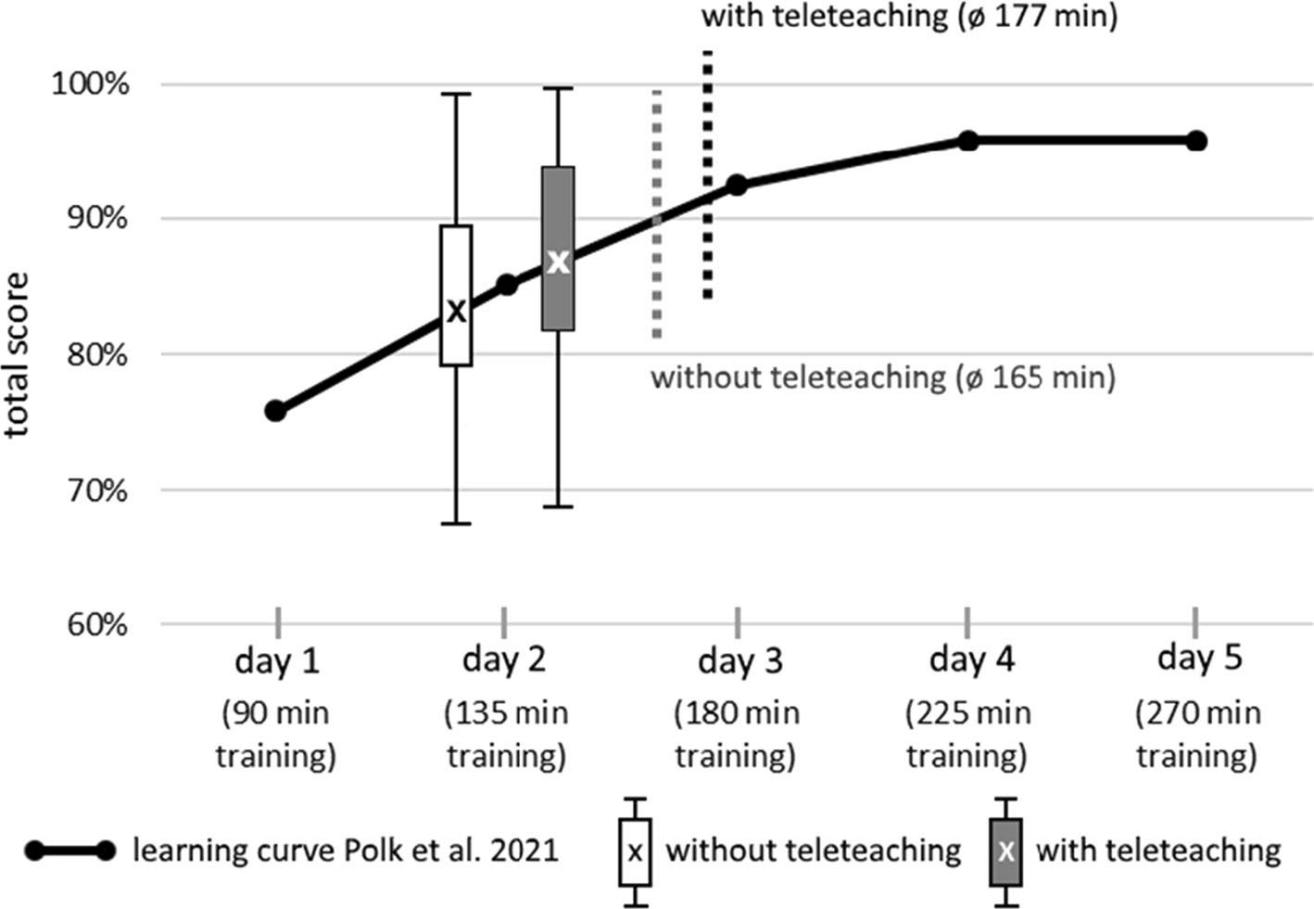
Comparison of the learning curve of presence teaching and digital teaching

The comparison of the digital teaching scores with the student’s learning curve for the CHNE by Polk et al. in 2020 [4]) is shown in Fig. 5. With respect to the achieved scores, the digital teaching groups were equivalent to about two teaching days (135 min training time) in presence. A tendency towards the performance after three presence teaching days was seen in the GwTT.

With respect to the students’ self-reported practicing time their expected performance on the learning curve would be significantly higher (Fig. 5).

Therefore, sole digital teaching (with and without teleteaching) leads to lower performances in the total score when compared to clinical teaching in presence.

## Discussion

The results provide a rational basis for evaluating the conversion from presence teaching to full-digital teaching in the context of a complex psychomotor skill, the CHNE. According to the results, it is possible to teach and assess a complex psychomotor skill completely digitally. By comparing the results of digital teaching with classroom teaching in presence, a validated statement can also be made about the influence of the different teaching models on learning success.

### Teleteaching has a significant impact on learning success

Data analysis revealed that teleteaching has a significant impact on the total score of the operationalized CHNE. The poorer performance of the GwoTT indicates that complex psychomotor skills can only be partially taught through sole self-study with a manual and instructional videos. Teleteaching and its possibility of direct interaction, feedback and question/answer significantly improves the performance in CHNE. Therefore, offering teleteaching can be considered as a quality feature of digital teaching [4]. In our case, teleteaching was done by an ORL specialist. In view of already published data [3], peer teleteaching by a qualified student tutor would expectably also be conceivable.

The evaluation of the subexaminations shows significantly better results in anterior rhinoscopy and laryngoscopy in the GwTT. Both laryngoscopy and anterior rhinoscopy are very demanding examinations in terms of manual skills, which are difficult to be mastered in self-study without feedback. By targeting correct execution and potential errors, teleteaching significantly improves the performance of these subexaminations. Significantly better results could be achieved with the presence of a teacher in form of teleteaching.

Also in otoscopy, oral inspection, posterior rhinoscopy, and general head and neck examination, the GwTT scored better. However, these differences are not significant. Perhaps the manual and instructional videos can better illustrate these subexaminations than for laryngoscopy and anterior rhinoscopy. Students may also be more familiar with oral inspection and general head and neck examination through other specialties, such as neurology, internal and general medicine.

### Comparison of learning success of digital teaching and presence teaching

In 2011, Kemper et al. [2] published total scores and scores of the subexaminations of the standardized CHNE during presence teaching. Using one and the same standardized checklist at the same medical faculty over the years, it is possible to compare the data of presence teaching and digital teaching in this study. Presence teaching was significantly superior to digital teaching with and without teleteaching in the total score and in all subexamination scores.

In 2020, Polk et al. [4] published the learning curve of CHNE of presence teaching. The presence CHNE course consisted of an initial demonstration by an ORL physician (45 min) followed by 5 days of training (90 min on day 1, 45 min each on days 2–5). An ORL physician performed the practical examination on day 5 [4]. Again, data collection was performed under identical conditions at the same medical faculty using the same operationalized CHNE and the same checklist. The GwTT achieved a total score equivalent to day 2–3 of the presence CHNE course. The GwoTT performed worse and achieved a total score equivalent to day 1–2 of the presence CHNE course.

These comparisons clearly show that students perform significant better with presence teaching. Nevertheless, the here described digital teaching was able to achieve a learning success, which is equivalent to about two days of presence teaching. However, teleteaching again enhances digital teaching results. Comparison of the data suggests that highly complex psychomotor skills such as CHNE can be taught more vividly and touchable in presence teaching than through digital teaching. In the future, hybrid models such as inverted classroom would be conceivable. Practical basic skills could be taught through digital self-study. Subsequently, targeted face-to-face teaching could mediate the deepening and broadening of practical skills in less time than presence teaching alone.

In 2021, Krauss et al. [5] published also data about the effectiveness of digital teaching of practical skills of CHNE. In daily videoconferences, a total of 146 students learned the CHNE during a period of five days. The learning curve was measured by analyzing a subgroup of 48 students on a daily basis. The assessment of the entire cohort was performed on the fifth day using a standardized checklist. The course structure is identical to the course described by Polk et al. [4]. During the 5 days of daily digital teaching, a significant increase in performance of all subexaminations was observed. Especially in laryngoscopy and posterior rhinoscopy, a very strong increase in performance was achieved through the daily digital teaching units. After 5 days, results of between 88 and 100% were achieved in all subexaminations. The authors conclude that practical skills of CHNE can also be acquired through digital teaching. Compared to the data published here, the daily digital teaching units lead to significantly better results. In addition, the daily digital course achieved comparable results as presence teaching. It can, therefore, be concluded that the expansion of digital teaching can achieve a further benefit in teaching practical skills.

### Correlation between practice time and total score is feedback dependent

The GwTT shows a significant positive correlation between practice time and total score. This underlines the importance of any face-to-face teaching, even in a digital manner. During a teleteaching session, students were able to ask questions, receive repeated demonstrations of subexaminations and got detailed and specific feedback by an ORL-specialist. In addition, common performance flaws were explained in anticipation. This feedback allowed students to improve their performance based on the amount of their invested practice time and prevented the consolidation of errors. Polk et al. [4] showed that in the presence CHNE course, overall performance increased steadily from day 1 to day 5 as a function of applied practice and teaching time.

The GwoTT shows a non-significant negative correlation between practice time and total score. The longer they practiced without feedback, the worse their overall performance became. Lack of feedback may have exacerbated individual errors in the performance of the CHNE over time as practice progressed.

When evaluating these correlations, it must be noted that students had to indicate the practice time independently. Therefore, this information is not verifiable, even though the exercise time distribution was comparable in both groups. For the here reported digital study population the average practice time was significantly shorter than in the usual presence CHNE courses. Here, the practice time was 270 min [4]. The reported average practice time for both digital groups in this study is equivalent to the practice time of 3 days in the regular presence CHNE course. The total score of digital teaching is rather close to the score of day 2 of presence teaching. Presumably, a further increase in practice time and/or teleteaching could lead to higher results and maybe reach mean scores of presence teaching. Nevertheless, sole digital teaching for clinical and technical skills is not desirable but the results presented here demonstrate the great potential of digital teaching formats and achievable results in self-directed learning.

### Digital assessment of psychomotor learning

A core component of university teaching in medicine is the teaching of psychomotor skills. CHNE represents a highly complex psychomotor skill. There are three types of exams in university teaching: oral, practical, and written. Psychomotor skills can only be adequately assessed through a practical exam. A digital exam is not an exam type, but an exam format [6]. The digital format must be able to assess the practical skills adequately in the way of a practical exam. Performance in digital examinations may not be fictitiously evaluated in the event of technical malfunctions or due to weaknesses of the digital format [6]. In this study, high-quality evaluation was ensured by excluding videos that could not be adequately evaluated due to camera perspective or light setting. In addition, the evaluation scale must not be changed by the fact that exams are taken digitally [6]. Due to the use of the already established checklist, the operationalized CHNE was assessed as in the days of presence teaching. This allows for direct comparison of results with respect to the teaching conditions as the variable factor.

The different examination setting in the present study must be taken into account. Digital exams can also be taken in real time and then reflect a live situation more accurately. Here, video recordings of the exam performance were sent in. These can be recorded several times until the best possible performance is achieved and then used. This possibility does not exist in real-time examinations (live as well as digital).

### Limitations of digital teaching

Critics say digital teaching in medicine should remain an exception. Complex scientific facts, psychomotor skills, and patient handling cannot be adequately taught through digital teaching [6, 7]. The elimination of presence teaching attacks key elements of university teaching. Digital teaching reduces direct interaction between students and teachers, the inclusion of the teaching environment, facial expressions and gestures, and individual rhetoric [8]. A survey conducted in the summer semester of 2020 found that 56.7% of German students wanted a return to presence teaching as soon as possible [9]. Technical problems, quality losses and limited didactics urgently need to be remedied by a strategic further development of digital teaching.

As teachers and authors, we share this opinion unreservedly. Nevertheless, the learning success through digital teaching in this study can be used to establish future hybrid courses in the sense of blended learning. In this case, the basics can be taught in digital formats and learned in self-studies, followed by in-depth instructions in presence teaching.

### Classification of digital teaching formats

The COVID-19 pandemic called for a rapid digitalisation of university teaching. Many teaching formats were created and offered completely digitally for the first time. In addition to the didactic, time and personnel costs, there is also a financial cost for the creation of digital teaching formats. The range of digital teaching formats is very diverse. Central elements of teaching and the immense challenge of digital teaching are the teacher's effort and the students’ ability to interact.

The teacher’s effort can be very variable in digital teaching. There is digital teaching material that is produced once and then made permanently available to the students. This requires a one-time effort on the teacher’s part. Live teaching, also when delivered digitally, has to be contrasted with this. It is closest to face-to-face presence teaching and requires the teacher to be present in real-time, even though the teacher may be physically distant from the students.

Face-to-face presence teaching is characterized by student interaction with the teacher. In digital teaching, there is often a physical separation between the teacher and students. Not every digital instructional format offers students the opportunity to interact with the teacher. However, interaction and discussion between instructors and learners is essential maximizing learning success. Thus, a distinction should be made between digital formats with and without opportunities for interaction.

Using a four-field table (Table 1), we aimed to classify digital teaching formats in terms of teacher’s effort and interaction opportunities.

**Table 1:**
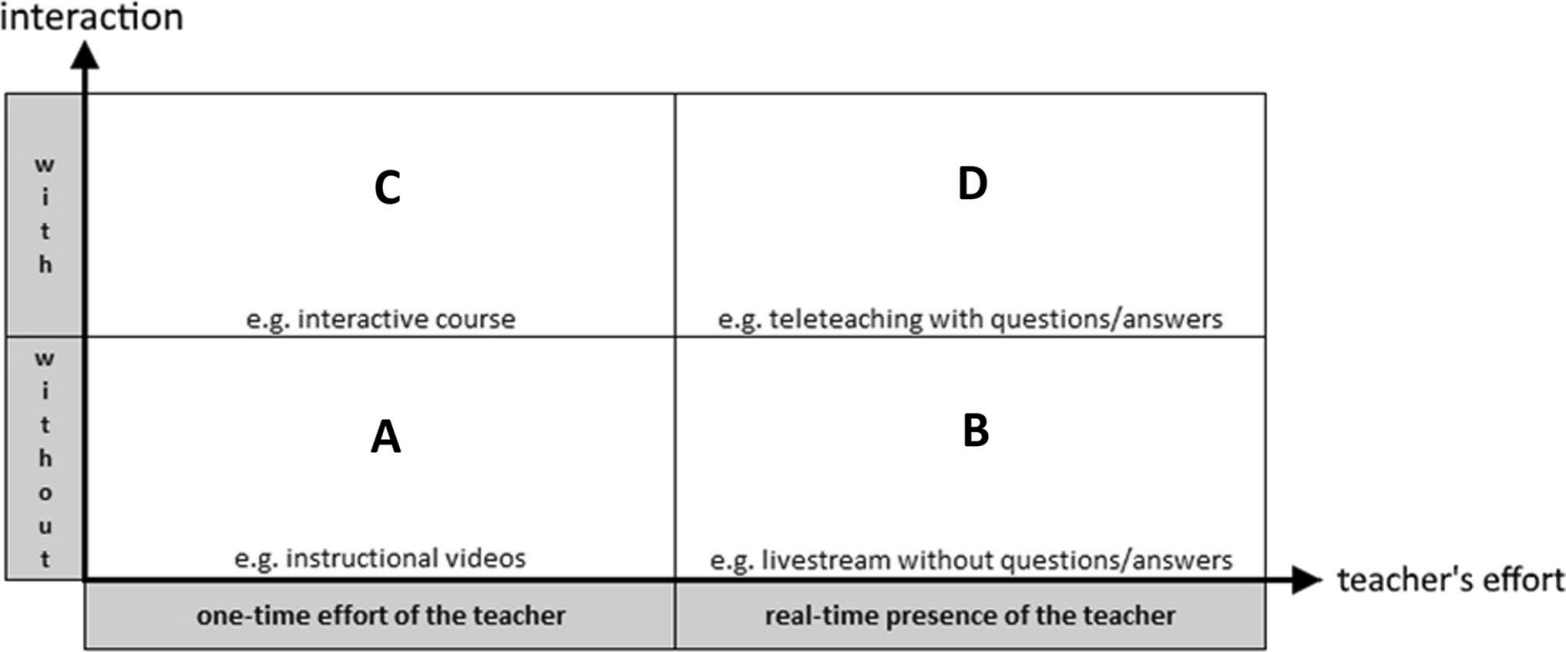
Classification of digital teaching formats

Category A contains one-time produced educational content that does not involve interaction opportunities. This includes the CHNE instructional video from this study.

Category B includes digital real-time teaching without interaction, such as a livestream of a surgery without the opportunity to interact, e.g. ask questions.

Category C represents digital teaching formats that are created once but allow for student interaction, such as an interactive student course without the teacher’s presence in real time.

Category D is closest to face-to-face presence teaching and requires the real-time presence of the teacher as well as student interaction. The CHNE-teleteaching used in this study is an example of this category.

This categorisation can be helpful in planning and evaluating one's own curriculum. Each teaching unit offered can be classified in one of the categories. Thus, the true teaching effort in the educational programme can be quantified and the amount of student interaction can be estimated.

### COVID-19 drives the curriculum and digitalisation

The digital transformation of German university teaching has been planned and discussed for years, almost a decade. This transformation was seen as a long-lasting disruptive process of change and innovation [10]. However, the COVID-19 pandemic became the driving force instead of didactic rationale or institutional strategy. With the elimination of presence teaching, medical faculties were forced to immediately convert teaching into digital formats [10–15]. Nevertheless, a survey of German ENT clinics and academic teaching hospitals revealed obvious discrepancies between available resources and effectively digital teaching and learning contents in the summer semester of 2020 [7]. Prerequisites for the sustainable digitalisation of university teaching are strategic and interdisciplinary processes as well as the creation of competence centres of the educational institutions [10].

## Conclusion

Due to the COVID-19 pandemic, an enormous and rapid digitalisation had to take place in order to maintain university teaching worldwide. Here, the complex psychomotor skills of CHNE were taught and assessed fully digitally for the first time. Due to the operationalized examination and the checklist-based practical examination, a comparison with the results of presence teaching was possible. The data collected are of enormous importance with regard to the objective assessment of learning success with exclusively digital teaching and testing of psychomotor skills. The significant impact of teleteaching on overall performance stresses the importance of interaction and direct feedback with the teacher. However, even with teleteaching, the learning success of psychomotor skills with presence teaching could not be achieved. The here presented results can be used for the further development of digital teaching and blended learning formats. If presence teaching is still not possible in the future, digital teaching can be expanded to maximize learning success of psychomotor skills. Expanding teleteaching with interaction capability could possibly bring it closer to presence teaching. A categorisation of digitally offered teaching units can help in planning and evaluating one's own curriculum. In this way, a targeted mix of teaching units can be created that is as close as possible to regular classroom teaching.

## Data Availability

The data that support the findings of this study are not openly available due to reasons of sensitivity and are available from the corresponding author upon reasonable request. Data are located in controlled access data storage at TU Dresden Faculty of Medicine and University Hospital Carl Gustav Carus, Department of Otorhinolaryngology Head and Neck Surgery, Dresden, Germany.
